# Toxins and the Liver1Read at the forty-second annual meeting of the Medical Association of Central New York, held at Auburn, October 19, 1909.

**Published:** 1910-06

**Authors:** William Mortimer Brown

**Affiliations:** Rochester, N. Y.


### Toxins and the Liver[1]

BY WILLIAM MORTIMER BROWN, M. D., Rochester, N. Y.

THE question of how great a degree of toxemia a given person may withstand and recover is one that furnishes a vast field for study and speculation, and any measure of accuracy which may be attained in the diagnosis of that dead line between the fatal and the recoverable cases of toxin sophistication of the blood stream, will indicate that the Utopian era of our knowledge of physiology and pathology of which we dream yet, may ere long be a more definite reality.

It is not our intention to bore you with the involved and somewhat vain theorising of the literature on this subject, and neither, on the other hand, will we be able to give you much definite information on a subject of which so few facts have been proven, but with your permission we will discuss very briefly one phase of the work that has been going on in the study of this general question of toxemia.

Until a comparatively recent time the study of puerperal eclampsia was conducted along lines relative to a renal injury, with a subsequent and resultant toxic condition due to the renal insufficiency, but post mortem findings, when properly studied, showed that in these cases other organs were the seat of definite pathological change, while in some of them there was an entire absence of kidney damage, and it began to dawn upon us that the renal injury was subsequent to and not etiologic of the general toxic condition and, further, that many times, coincident with or prior to the renal injury, there were changes in other organs which were in some manner the result of the toxic contamination of the blood current. One of the principal organs so affected is the liver and it is with especial reference to that and the effect of blood poison upon it that I ask your attention.

When we remember the double function of the liver, that of preparing food products for assimilation and also rendering innocuous the products of intestinal putrefaction and general body waste, we can readily see the importance of maintaining the functional integrity of that organ. Mohr and Freund have isolated

1. Read at the forty-second annual meeting of the Medical Association of Central New York, held at Auburn, October 19, 1909.

from the placenta certain hemolytic substances that are capable of producing hemolysis of human blood cells. Last October (1908) Welch of New York, published a study of the autopsy records of patients who died in the New York Lying-in Hospital from eclampsia. Let me quote: "Most of these cases show considerable hemolysis....The condition found in the parenchyma cells indicate the action of some dissolving poison. This agent is also capable of attacking the endothelium of the bloodvessels."

Granting the accuracy of these observations it is very easy to understand how the liver receives its injury in some cases, even to the extent of multiple hemorrhages and necrosis; but, on the other hand, is it so easy to follow it up and say what causes the profound nervous explosions of eclampsia or the death of the patient. Is it those same toxins that have come from the placental cells and have already caused a cellular autolysis in the liver, or are the various constitutional evidences of this disease, the direct result of the previous changes, that have taken place in the liver? and have thus deprived it of its power to defend the general cell metabolism? "It has been determined that normal cell metabolism is performed by means of enzymes, and if for any reason these enzymes become disturbed an autolysis results."

Williams, of Baltimore, called attention to two forms of necrosis in the liver, or at least that the cell destruction sometimes took place in the center of the lobule, while at others it invaded the lobule from the periphery and was produced by a thrombosis of the smaller portal vessels. He claimed that the central form of necrosis is associated with toxic or pernicious vomiting, while the peripheral variety is found in cases of eclampsia, and he believed that there were two different poisons which were associated each with its own disease.

Welch's report showed that in some cases of central lobular hemorrhage eclampsia obtained, while at times the peripheral form of necrosis was associated with toxic vomiting. It would seem to the writer that it is a doubtful advantage to go to the autopsy table for a diagnosis of pernicious vomiting or eclampsia. Of vastly more importance is the question whether this liver injury is in and of itself a fatal element or merely a symptom in these cases. Does the patient die because of the injury to the liver and the consequent loss of function; or is the autolysis in the liver merely an indication of the virulence of the toxemia?

Until case IV came under his observation the writer was inclined to think that when unmistakable signs of hepatic defect were present, as shown by the slow pulse, more or less icterus, bile in the urine, and general torpor and vomiting with eclampsia, a fatal ending was inevitable, but why may we not have various grades of degeneration in these cases and if recognised early enough and before too much damage has obtained, may we not avoid serious results in many cases? Fortunately the signs which we have been accustomed to class as evidence of a kidney defect, are also indicative of a deficient metabolism within the liver, but there are also many signs of decreased functional activity of the liver which antedate the graver symptoms. The realisation of the important part which the liver plays in maintaining a normal balance of health during pregnancy, adds emphasis to the importance of careful and painstaking observation of our patients with especial reference to the digestive tract.

The ascertaining of the relative amount of nitrogen which is being put out as urea, is of distinct value in determining a question of hepatic insufficiency, but that value is intensified when the percentage of ammonia nitrogen is also fixed. We have then the data which may establish with certainty the question of a more or less severe hepatic paralysis, and this disturbed ammonia urea relation may be present for a considerable time before the other signs appear. Although the exact percentage of ammonia increase may not be so important as Williams seems to believe, yet its general importance as a possible indicator of conditions precedent to an intoxication may be very great.

I have selected the histories of four patients that seem to me to illustrate the points that we have had under discussion. Case I. belonged to a class which, by all the laws of the literature on this subject, should have died and having died the liver should have shown multiple hemorrhages with central necrosis of the lobules, but she did not die. Case II. was a typical case of eclampsia without signs of liver injury, which recovered. Case III. will be the history of a toxemia of the hepatic type with the classical signs of liver involvement who died and the post mortem findings verified the diagnosis. Case IV. will recite the story of a case quite similar to Case III. but which, contrary to rule, did not die.

Case I.—Mrs. F., age 21, white, a native of England; in her second pregnancy. She relates that in her first pregnancy she often vomited blood, took but little food, had chills and fever, great weakness, and the like. She had a long, hard labor and was badly lacerated. The child died when less than a year old. She has never had any miscarriages. The present pregnancy began about January 25, 1906. In April she came to the United States having a very stormy voyage and was seasick all the way over. On May 7, she felt something warm in her throat and blood ran out of her mouth. There was no cough but the hemorrhage was followed by vomiting and diarrhea and a severe epigastric pain which was not affected by eating. The vomiting continued while under the care of her local physician who exhausted his resources without avail.

She entered the hospital July 15, with a diagnosis of pernicious vomiting, and for the purpose of having the pregnancy terminated. She was badly emaciated, unable to retain any food or medicine administered by mouth. The temperature was 100.5 degrees F. and pulse over 150. There was a faint trace of albumin. Williams has said that a patient who presents such a picture is in grave peril as the rising temperature and failing pulse antedate death but a short time, and that the only chance of recovery is an immediate evacuation of the uterine contents.

After due consideration it was deemed advisable to try and get her into a better condition before operating. Strychnine gr. 1|30 was given subcutaneously every four hours and the rectum was thoroughly emptied, first with a simple enema and then with a high gas enema. A severe epigastric pain was somewhat relieved by morphia gr. 1|8. She responded promptly to the stimulation the next morning her pulse being down to 120 and her temperature was 99°. After a rest of twenty-four hours she was given a thorough rectal irrigation of normal salt solution, followed with a nutritive enema of peptonised milk every three hours. The enemata were well retained and nothing whatever was given by the mouth for five days. After that, small quantities of peptonised milk were given, alternated with albumin water, which was well retained and from which time her recovery was very satisfactory. She was discharged from the hospital July 29.

Case II.—Miss B. White, age 24, II-para; previous pregnancy was normal but the delivery was instrumental because of uterine inertia, after twenty-four hours of labor. The history of the present pregnancy is vague, except that she has had a great deal of headache and edema of the lower extremities, with physical and mental lassitude. She entered the hospital May 8, with a history of severe epigastric pain and vomiting since the day before. She was restless and slept only a few moments at a time. The laboratory sent back a negative report on the urine. The vision was clouded and the arterial tension was above 190. Magnesia sulphates was administered together with a large quantity of fluid and the diet was restricted, especially the proteid element. The epigastric pain and the cloudy vision continued. On May 10, the blood pressure was 170, and an examination of a twenty-four hour specimen of urine showed a large amount of albumin and fine granular casts; the chlorides 3.6 and the total nitrogen 12.3, while the urea was only 7.6. With a limited diet and a free washing of the circulation her condition improved so that on May 15, the arterial tension was down to 133, and the urea elimination was up to 25.4 grains. A normal delivery occurred May 16, after a two hours' labor.

Case III is the only case that I am able to present with an autopsy record, but it is interesting as the case presented the clinical signs of eclampsia of the hepatic type and the post mortem findings verified the diagnosis.

CASE III.—Age 25, unmarried, primipara. About thirty weeks pregnant. No previous history of the condition was obtainable as her parents did not know she was pregnant, and she was in a semiconscious condition when she was admitted to the ward August 26. She had had several convulsions before she reached the hospital, and one just before delivery. When I first saw her she was only partly conscious and of a sallow color. Temperature normal, the pulse was only fifty-two and not strong. The area of liver dulness was somewhat diminished.

With this picture before me I ventured to predict a fatal termination, believing that we had a case of acute lobular necrosis in the liver, and that puerperal eclampsia with such hepatic injury is always fatal. The uterus was rapidly emptied and the circulation was washed as thoroughly as consistent with her condition. Although she had no more convulsions she remained in a torpid condition and died within twelve hours from the time of delivery. Autopsy was made August 27, about fourteen hours after death, and reported in the record as follows:

M. H. Age 25. Eclampsia. Abdominal viscera alone examined. Abdomen soft, no marked distension; peritoneal cavity contains a fair amount of free peritoneal fluid; stomach distended; intestines contain only a very moderate amount of gas; not injected. Liver weight, fifty-two ounces, pale yellowish in color, surface smooth and mottled with smaller and larger areas of dark red color. Cut surface pale with areas of congestion, corresponding to the dark mottling seen on the surface. Kidneys normal in size; no adhesions; capsules strip easily leaving surface of the kidney smooth. Surface pale with some very fine points of injection; on cut surface pale with some of the pyramids congested. The pathologist reports, after examination of stained sections, that the kidneys are the seat of acute parenchymatous nephritis and that the liver is the seat of extensive focal necrosis.

I should like to give Case IV with more detail because it developed into what appeared to be as definitely hepatic in type as the other, but seemed to prove that all cases of eclampsia with liver injury do not die.

CASE IV.—Mrs. K., age 20, white, Russian, primipara. Previous history not obtained, because the patient was delivered at home and was admitted to the hospital in a severe eclamptic condition. She entered the ward June 22, at ten thirty in the morning; pulse 124, temperature 103.4° F. It was stated that the child was born at ten o'clock the night before without attendance, and that at midnight she had some sort of spasm which was repeated at intervals until eight o'clock, when a physician was called who sent her to the hospital. It is not known how many convulsions she had before admission, but during the afternoon she had seven seizures at intervals of about an hour and lasting from one-half to three minutes, followed by stupor. The pulse was of fair quality but a variable rate, from 90 to 150!

arterial tension 150. During the night she was very restless but no convulsions and the pulse varied from 110 to 130. Was less stupid but irrational and had to be restrained.

June 23.—Very restless during the morning; pulse 78 to 108 and the tension 138. Quieter at night and appeared somewhat more rational, pulse 96 to 100. Patient seemed slightly icteric on the 24, but became more restless and uncomfortable in the afternoon. Irrational and got out of bed three times and appeared stupid; pulse 90, good quality. In the night she was restless, complained of headache and appeared to be dazed; pulse slow and full rate 58. Quite sallow, area of liver dulness much diminished.

June 25.—Restless with headache in the afternoon, pulse 60. During the night was restless, appeared dazed, complained of headache and pain in the epigastrium. Tried to get out of bed. June 26.—Was restless and delirious. Pulse weak 53. Nauseated and vomited greater part of the night. June 27.—Patient appeared to be in stupor. Does not respond to questions. Unable to retain medicine or nourishment. Pulse slow and weak. At night restless and delirious; nausea and vomiting. Headache, pulse 64 to 68.

At this time a blood examination showed 6,000 000 red cells hg. 90 per cent., color intex, .75 and a gross white count of 20 000. June 29.—Patient has been very irrational; restless and noisy most of the day. Dislikes the baby and complains of severe pain in the head and epigastrium; pulse 56 to 70. At night was extremely noisy and restless. Vomited ten ounces of coffee ground material. June 30.—Still restless and noisy; headache and vomiting; pulse 60. July 1.—Restless and noisy during the day but became more rational and quiet at night. Pulse 68. July 4.—Patient had a fairly comfortable day; pulse 70 and from this time she rapidly convalesced.

For the sake of brevity I have omitted the treatment of this case from the report, but in a general way it consisted of a thorough washing of the general circulation by means of magnesia sulphatis and giving large quantities of fluid, while relieving the liver of all demands possible by restricting the diet and giving intestinal antiseptics. Small amounts of morphine were given when necessary because of restlessness. At no time was the urine less than 1018 sp. gr. and only three days was there any albumin excreted. During the activity of her disease there was no secretion of milk but July 5, as she improved, lactation began and she was able to leave the hospital nursing her baby.

It will be noted that the blood picture of the last case showed some evidence of hemolysis, although the pathologist was not very clear on that point, and surely the clinical picture is that of one of the most profoundly toxic conditions, in which there was evidently quite extensive injury to the liver which developed on the fourth day, as evidenced by the sudden drop in the pulse rate which is quite characteristic of cases of hepatic insufficiency.

Whether the autolysis in the liver was associated with cellular degeneration in other parts of the body, we are unable to say, for it is still uncertain whether the autolysis results directly from the action of the hemolytic poison from the placenta or indirectly from the perversion of enzymes, due to the hepatic defect.

666 East Avenue.

